# Ecological aesthetic assessment of a rebuilt wetland restored from farmland and management implications for National Wetland Parks

**DOI:** 10.1371/journal.pone.0223661

**Published:** 2019-10-10

**Authors:** Mingyang Sun, Xue Tian, Yuanchun Zou, Ming Jiang

**Affiliations:** 1 Jilin Provincial Joint Key Laboratory of Changbai Mountain Wetland and Ecology, Northeast Institute of Geography and Agro-ecology, Chinese Academy of Sciences, Changchun, Jilin, China; 2 College of Art & Design, Jilin Jianzhu University, Changchun, Jilin, China; Beijing Forestry University, CHINA

## Abstract

While wetlands are usually used as a natural approach to remove biodegradable pollutants in surface water, their purification efficiencies coupled with their aesthetic features are of less concern. The water quality, plant landscape, acoustic environment and odour indicators were investigated in the surface water inlet and outlet of the Fujin National Wetland Park (FNWP), restored from farmlands in Northeast China. Major concentrations of pollutants in the inlet and the outlet subjected to surface flow wetland treatment were monitored, and the removal efficiencies were calculated based on 54 water samples (6 sites×3 seasons×3 replicates). The results showed that the total nitrogen (TN) and organic carbon in surface water decreased significantly after the wetland treatment, while the total phosphorus (TP) did not decrease significantly. The removal efficiencies for TN and BOD_5_ changed seasonally and reached 69.08% and 60.44%, respectively. An ecological aesthetic index (EAI) was developed based on the trophic state index coupled with plant landscape, acoustic and odour indicators, and the calculated EAI showed that the outlet delivered a more aesthetically harmonious appearance than the inlet in spring and autumn, but not in summer. Based on the current aquatic macrophyte species and documented purification efficiencies in FNWP, we recommend an improved ecological aesthetic management approach that utilizes and arranges diverse native plants from the surrounding wetlands (e.g. *Scirpus validus*) in addition to local *Nelumbo nucifera*, *Nymphaea tetragona* and *Myriophyllum spicatum*, and conserves the indicative and endangered species (*Aldrovanda vesiculosa*), from the visual appeal of the waterscape.

## Introduction

Wetlands provide some of the most important ecological functions, such as providing nursery areas for fish, supporting an abundance of wildlife, controlling flooding, and filtering nutrients, sediments and even some harmful pollutants [1]. Wetland plants have different categories of size, form and species. There are, for example, floating, emergent, and submerged plants of the littoral zone [2]. Aquatic plants have also been used successfully as a bioremediation method for purifying surface water [3–5]. Moreover, environmental pollution in surface water is mitigated by using aquatic plants that conserve ecological form and purification function as biodegradation media [6–11].

China is one of the largest agricultural countries in the world, with rich agricultural resources, and the majority of the population resides in rural areas. At the beginning of the founding of the People's Republic of China, to solve the problem of food and clothing for the population, people occupied a large area of primitive forests and wetlands for agricultural production. With the development of the country, to strengthen the construction of an ecological civilization, China has successively issued policies for restoring farmlands to forests, grasslands, wetlands and other ecologically sound lands. Since 2014, some specific policies related to restoring farmlands to wetlands, such as Wetland Ecological Benefits Compensation and Wetland Protection Rewards, have been piloted with the increased allocation of forestry subsidy funds from the central government [12].

In recent years, the restoration of farmland to wetlands as an ecological political policy has been a matter of growing concern in China. A major project of further farmland being restored to wetlands is in full swing in China's north-eastern Heilongjiang Province [13–16]. In the course of this process, surface water treatment is typically a chief concern. Surface water treatment becomes the management focus for any newly created or rebuilt wetland. This is due to the need to control the levels of pollution from the surface runoff that may be contaminated by chemicals in fertilizers, pesticides, animal slurry, crop residues or irrigation water from the former farmland. Mitigation and removal of these pollutants by biological approaches is the main focus of the ecological restoration and rebuilding practices within this industry.

**Fig 1:**
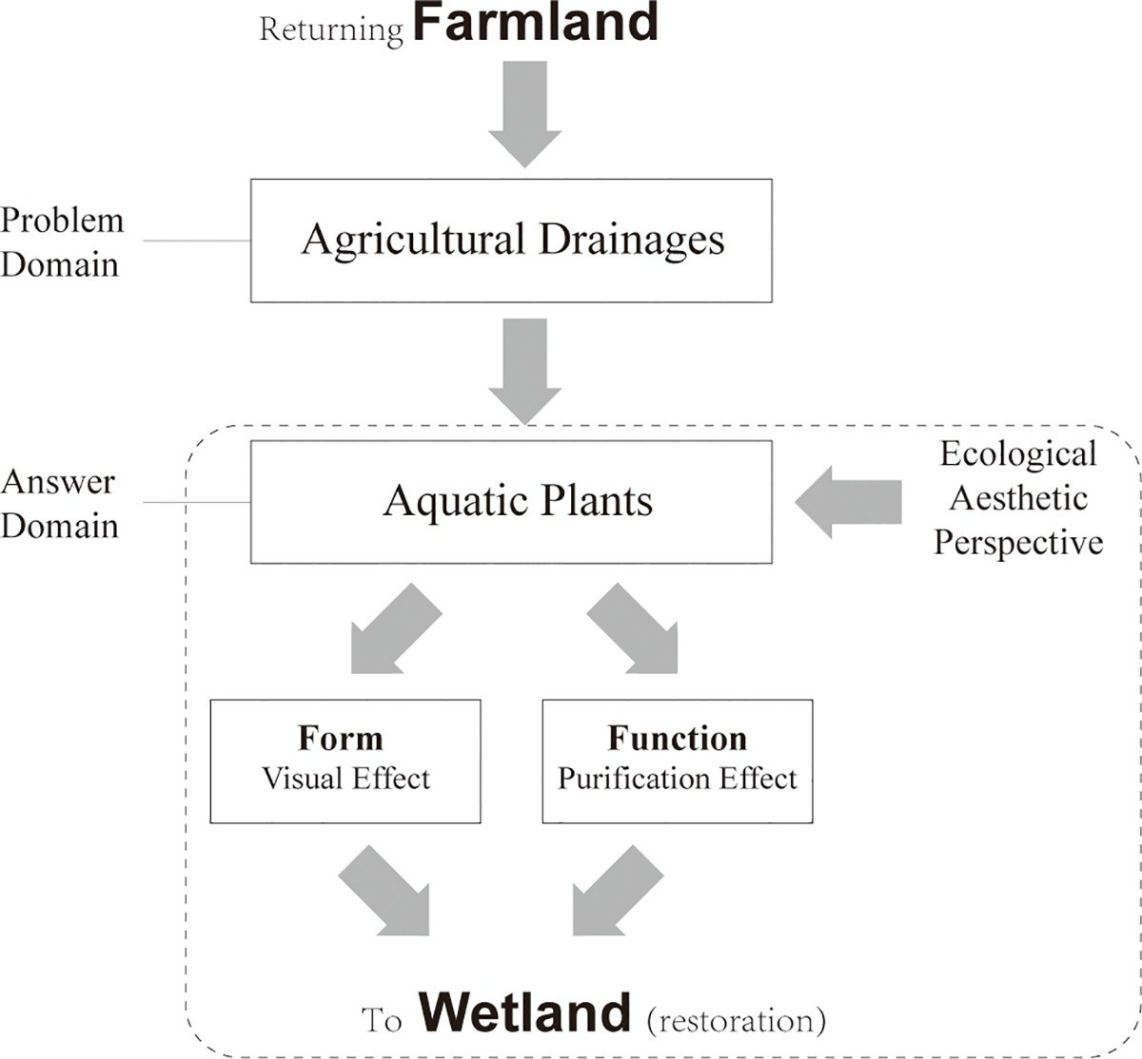
Problem and answer domains of restoring farmland to wetland. The objective is a workflow that shows the problem domain (agricultural drainage) and the answer domain (aquatic plants) from farmland to wetland. During this process of wetland restoration, aquatic plants are the biotechnological medium for removing pollutants (function) while improving the beauty (form) of FNWP from an ecological aesthetic perspective.

Generally, wetland management aims to preserve or restore desirable ecological characteristics or functions [17, 18]. It is now well recognized that some social criteria should also be included in these goals. China’s restoration of farmland to wetlands is mainly concentrated in the protected areas of wetland parks. In addition to the water quality protection target, there are also the functions of protection for education and ecotourism [19,20], which necessitate aesthetic concerns and improvements. New thoughts or methods are required to combine the art and science perspectives [21]. There is an urgent need to develop long-term, biological, self-sustaining systems to purify surface water during the process of restoring farmland to wetlands in China (Fig 1).

In this paper, we explored the effects of aquatic plants on surface water quality through an ecological aesthetic perspective. The objectives of this paper were (1) to investigate the seasonal surface water purification efficiencies of a typical rebuilt wetland returned from farmland; (2) to assess their ecological aesthetic index (EAI) based on water quality coupled with plant, sound and odour indicators; and (3) to improve the holistic perception effect of the wetland park to cater to the demands of individuals with an aesthetic perspective.

## Materials and methods

### Study area

**Fig 2:**
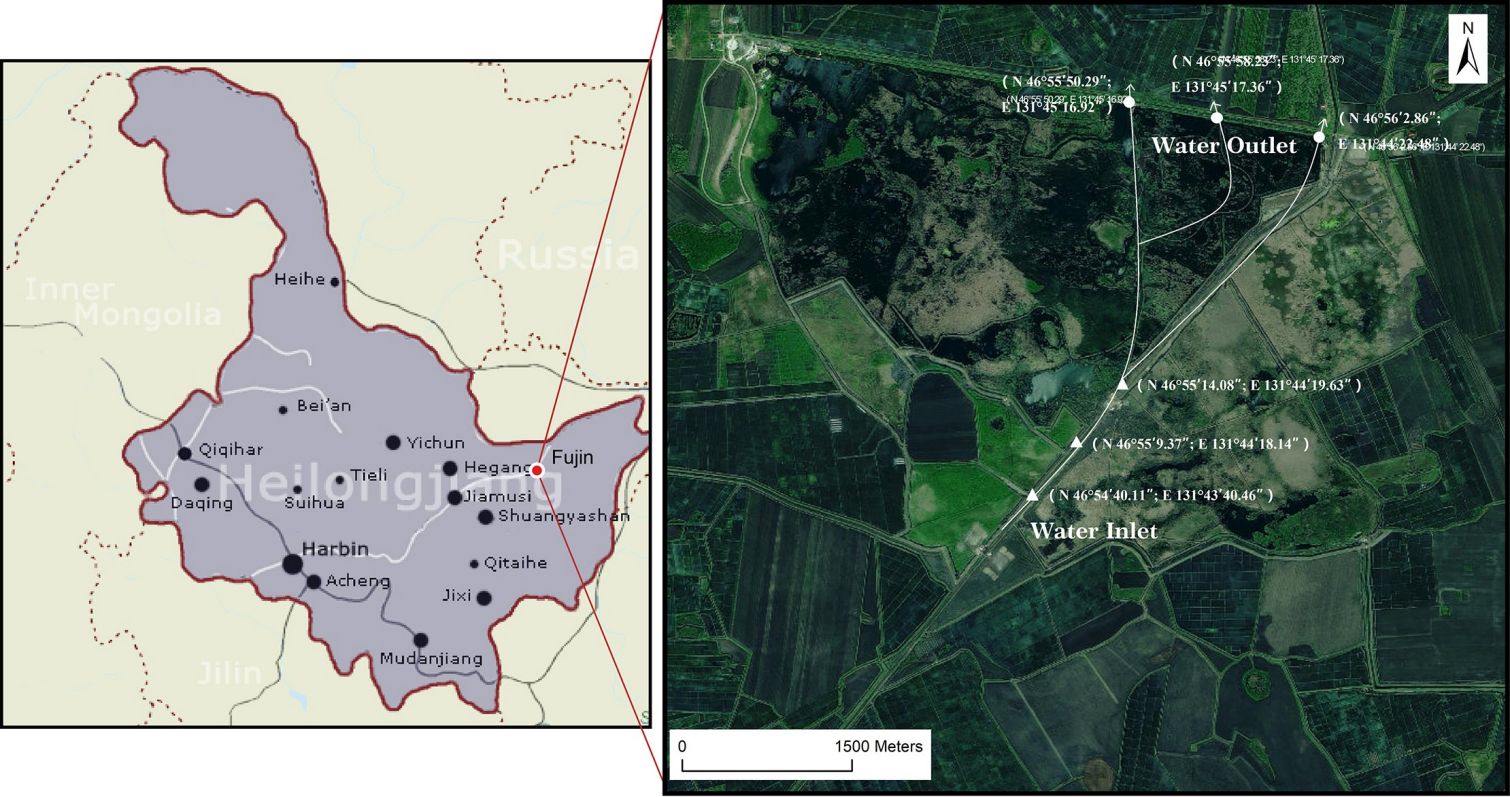
Location and monitoring sites of the Fujin National Wetland Park (FNWP).

The Fujin National Wetland Park (FNWP, 46°55′52.72″N,131°44′51.33″E), located in Fujin City, Heilongjiang Province, has a total area of 2200 ha(Fig 2). More than half of FNWP is rebuilt wetlands from occupied cultivated farmlands since 2009. The wetland is supplied by upstream surface water and precipitation, with the main water flow from southwest to northeast. The annual average temperature is 2.5°C, ranging from -20.4°C in January to 22.2°C in July. The annual precipitation is 609 mm, with the most monthly precipitation from May to September. The frozen period is from November to April of the next year. The previous study showed that the surface water within the wetland had a light eutrophication status [22, 23].

### Surface water sampling

Wetland surface water samples were collected at the inlet and outlet on 27 May (spring), 30 August (summer) and 30 October (autumn) of 2017. This survey had been granted the permission from the Administrative Bureau of the FNWP. In addition, such studies did not involve endangered or protected species. The three replicated samples in the inlet were along the former ditch from the wetland flooding boundary with 500 m intervals between each site. The three replicated samples in the outlet were collected in the same way from the gate (Fig 2). Water transparency was measured as Secchi depth (SD) using a Secchi disk after water sampling.

### Plant selection based on ecological aesthetic approaches

According to a survey of FNWP, there are five types of wetland classification by aquatic vegetation in FNWP. These include reed (*Phragmites australis*)wetland, cattail (*Typha angustifolia*) wetland, submerged and floating plant wetland, wet meadow, and open water (Fig 2). The cattail wetland is the largest, with an area of 319.8 ha, and accounts for 31.2% of the total wetland area. The reed wetland has an area of 256.3 ha and accounts for 25.0% of the total wetland area. The rest plants were distributed sporadically without accurate survey. The area of submerged and floating plant wetland was greater than that of open water. The dominant aquatic plants growing in FNWP are listed in Table 1.

**Table 1:** Dominant aquatic plants for purifying agricultural drainage in the Fujin National Wetland Park (FNWP).

Vegetation type	Botanical name	Purification effect	Reference
**Emergent plants**	*Phragmites australis*	COD, BOD_5_, TN	[24]
	*Typha angustifolia*	TN, TP	[25]
	*Polygonum amphibium*	TN	[26]
	*Lythrum salicaria*	TN, TP	[27]
	*Nelumbo nucifera*	TN, TP, COD, TDS	[28, 29]
**Floating plants**	*Nymphaea tetragona*	TN, TP	[30]
	*Nymphoides peltata*	Cd	[31]
	*Lemna minor*	TN, TP	[32]
**Submerged plants**	*Potamogeton pectinatus*	TN, TP	[33]
	*Potamogeton crispus*	TN,TP, TSP	[34]
	*Myriophyllum spicatum*	TP	[35]
	*Hydrilla verticillata*	TP	[35]
	*Aldrovanda vesiculosa*	-	[36]

### Chemical measurements

The total nitrogen (TN) and total phosphorus (TP) in water samples were measured using a continuous flow analyser (Skalar, the Netherlands). Chemical oxygen demand (COD_Mn_) was determined by a permanganate index. Biochemical oxygen demand (BOD_5_) was determined by the dilution and seeding method. Chlorophyll-a was measured by the spectrophotometric method after extraction by acetone. The specific processes were referred to standard methods for observation and analyses of water environment factors in Chinese Ecosystem Research Networks [37].

### Ecological aesthetic assessment

The EAI assessment system was designed based on Carlson's trophic state index (TSI)coupled with plant landscape, acoustic and odour indicators (Table 2)[38]. The seasonal EAI values of the wetland surface water inlet and outlet were calculated as the weighted sum of TSI, a plant landscape visual hierarchy indicator (PLVHI), an acoustic environmental indicator (AEI) and an odour indicator (OI).PLVHI, AEI and OI were expressed as the means of tourists’ personal experiences by questionnaire. The weights of the indicator were obtained based on three steps. First of all, making a questionnaire with Likert scale, the EAI user survey for ecological survey for design and construction. Secondly, some locals and tourists who are reprehensive according to the age, gender, and occupation and so on are invited to do the questionnaire in different seasons as a reference. Finally, some experts from design, environment, and ecology evaluate the questionnaires again from their academic perspective. There are two aspects of evaluation, one is from the perspective of landscape ecology, evaluate objectively water quality, the other is from the perspective of landscape aesthetics, evaluate subjectively (human perception system). The effect of water landscape is pleasant or unpleasant based on both sides. Each of them interact each other in relative range.

**Table 2:** Assessment criteria of the ecological aesthetic index (EAI) that is calculated as the weighted sum of the trophic state index (TSI), the plant landscape visual hierarchy indicator (PLVHI), the acoustic environmental indicator (AEI) and the odour indicator (OI).

TSI	PLVHI	AEI (daytime, dB)	OI	Level	EAI	Holistic Perception Effect
**Oligotrophic**	Well organized	Very peaceful <20	Sweet-smelling	I	0–30	Harmonious
**Mesotrophic**	Ordered	Tranquil 20–40	Fragrant	II	30–50	Good
**Eutrophic**	Irregular	Serene 40–60	Pleasant	III	50–60	Comfortable
**Supereutrophic**	Disordered	Noisy 60–70	Terrible	IV	60–70	Unacceptable
**Hypereutrophic**	Chaotic	Very noisy >70	Malodourous	V	>70	Bad

### Statistical analysis

Statistical analysis was performed using SPSS Statistics for Windows, Version 21.0 (IBM Corp., USA). Repeated-measures analysis of variances (ANOVA) was used to analyse the interaction effects of season and sampling location. Mauchly's Test of Sphericity was first examined, and Greenhouse-Geisser adjustment was adopted when the null hypothesis was rejected. Two-way ANOVA was used to compare the means of TN, TP, COD_Mn_, BOD_5_, chlorophyll-a, SD and BOD_5_/COD_Mn_ in the inlet and outlet of different seasons. All means and standard errors of data were calculated using Origin Pro 8.0 (Origin Lab Corp., USA). The water quality graphs were drawn by Origin Pro 8.0, and the others were drawn by Photoshop CS 5 (Adobe Inc., USA).

## Results

### Seasonal surface water qualities of the inlet and outlet

According to the repeated-measures ANOVA, the interaction effects of season and sampling location were only significant for TN and BOD_5_. COD_Mn_, Chlorophyll-a and SD only significantly changed with the seasons, while the changes in TP were non-significant (Table 3).

**Table 3:** Repeated-measures analysis of variance (ANOVA) of wetland treatment on the total nitrogen (TN), total phosphorus (TP), chemical oxygen demand (COD_Mn_), biochemical oxygen demand (BOD_5_), chlorophyll-a and Secchi depth (SD).

Variable		TN	TP	COD_Mn_	BOD_5_	Chlorophyll-a	SD	BOD_5_/COD_Mn_
**Season**	*F*	116.168	0.076	12.868	50.993	16.279	17.238	27.877
	*P*	<0.001	0.927	0.002	<0.001	0.002	0.001	<0.001
**Season× location**	*F*	10.728	2.394	3.967	12.128	3.714	2.952	5.722
	*p*	<0.001	0.107	0.062	0.001	0.072	0.110	0.027

**Fig 3:**
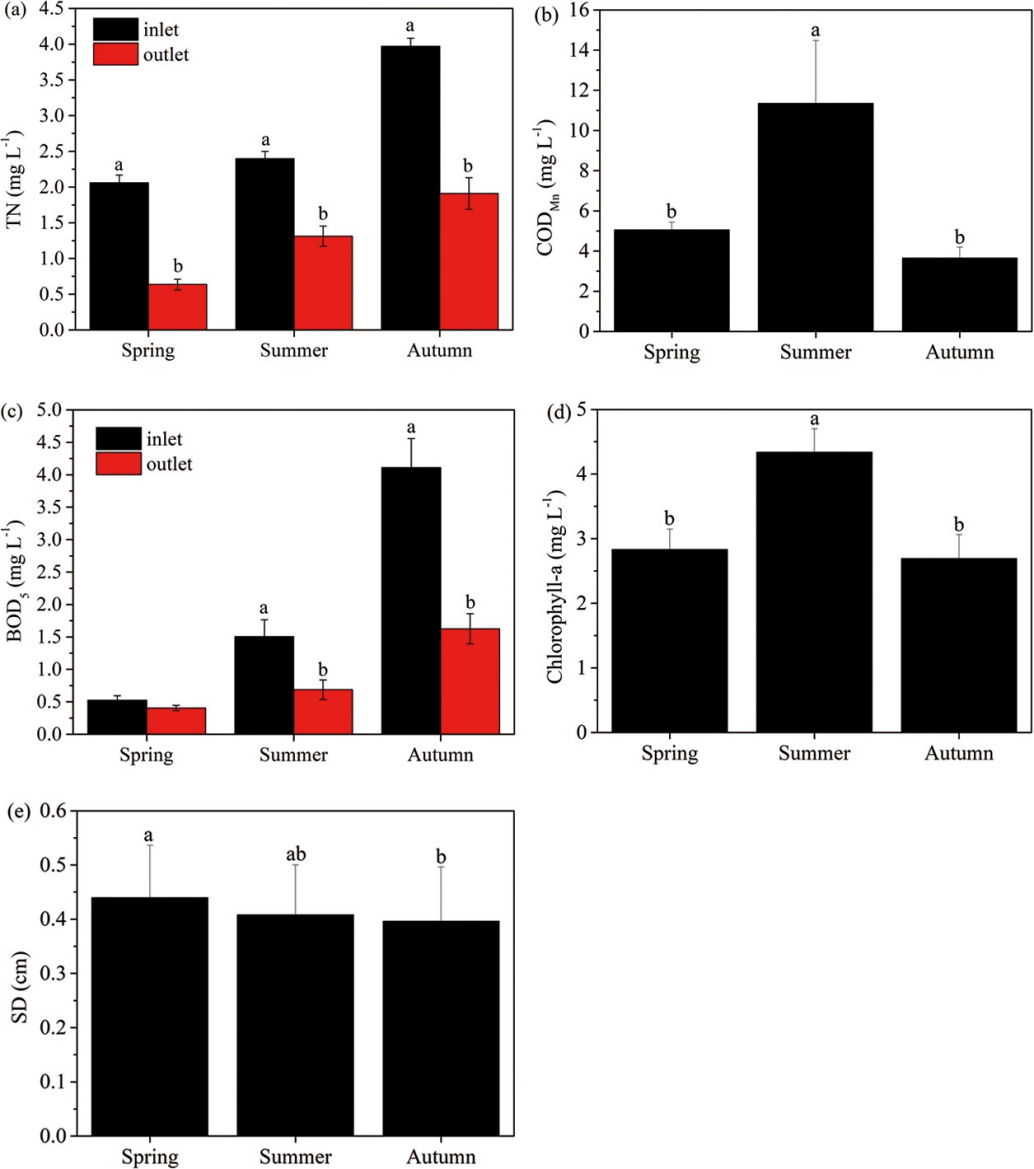
Seasonal variations in total nitrogen (a, TN), chemical oxygen demand (b, COD_Mn_), biochemical oxygen demand (c, BOD_5_), chlorophyll-a (d) and Secchi depth (e, SD) in the wetland.

TN in both the inlet and outlet increased from spring to autumn, and the former was always greater than that of the latter (Fig 3A). The mean TP was (0.10±0.02) mg L^-1^. COD_Mn_ and chlorophyll were the highest in summer (Fig 3B and 3D). BOD_5_ showed similar trends as TN, but there was an insignificant difference between the inlet and outlet in spring (Fig 3C). SD decreased from spring to autumn (Fig 3E).

### Major pollutant removal efficiencies

**Fig 4:**
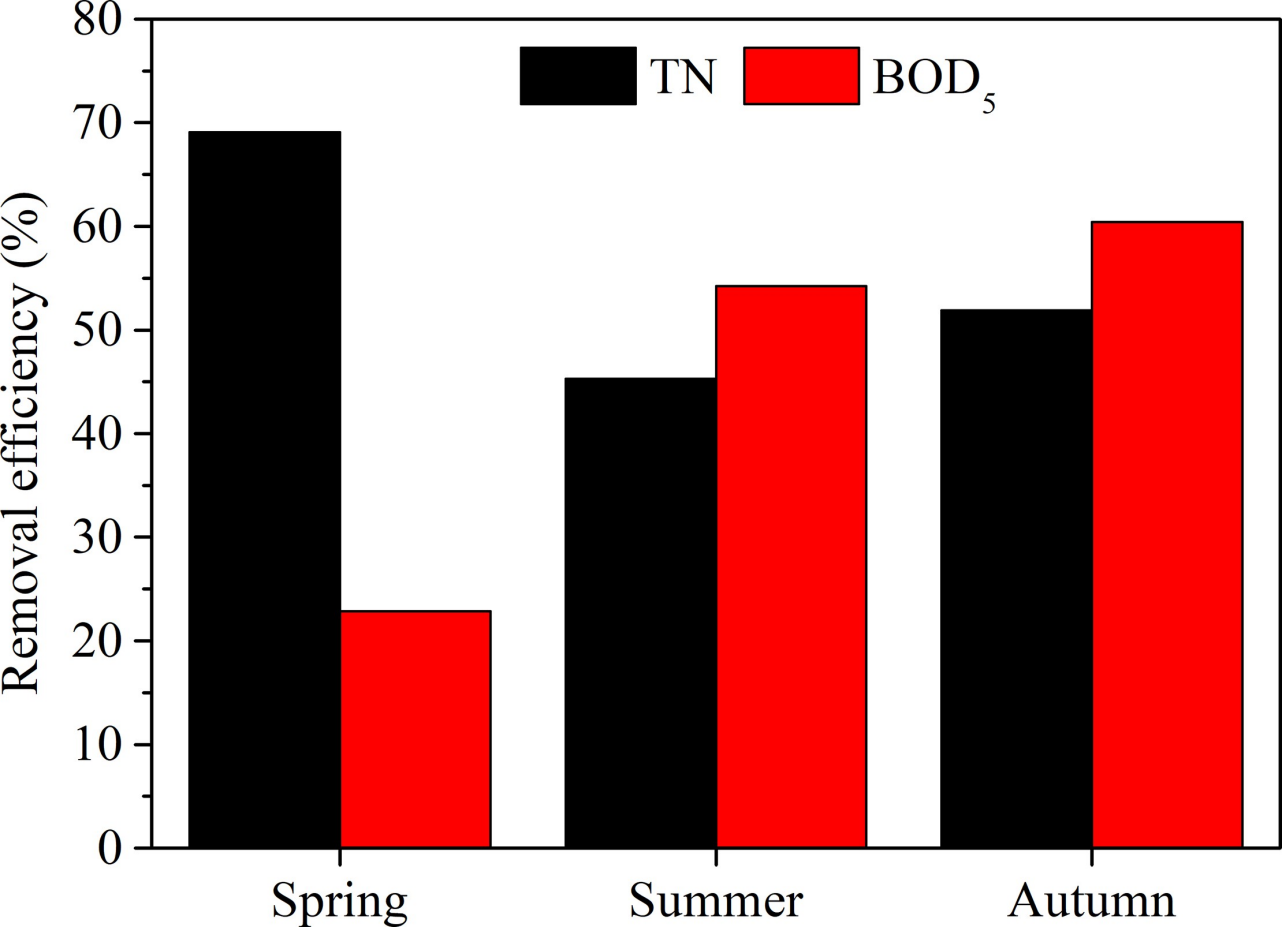
Seasonal variations in removal efficiency for TN and BOD_5_.

The removal efficiency for TN and BOD_5_ changed from 45.31% to 69.08% and from 22.83% to 60.44%, respectively. The removal efficiency of BOD_5_ increased from spring to autumn, while that of TN first decreased firstly from spring to summer and then increased in autumn (Fig 4).

**Fig 5:**
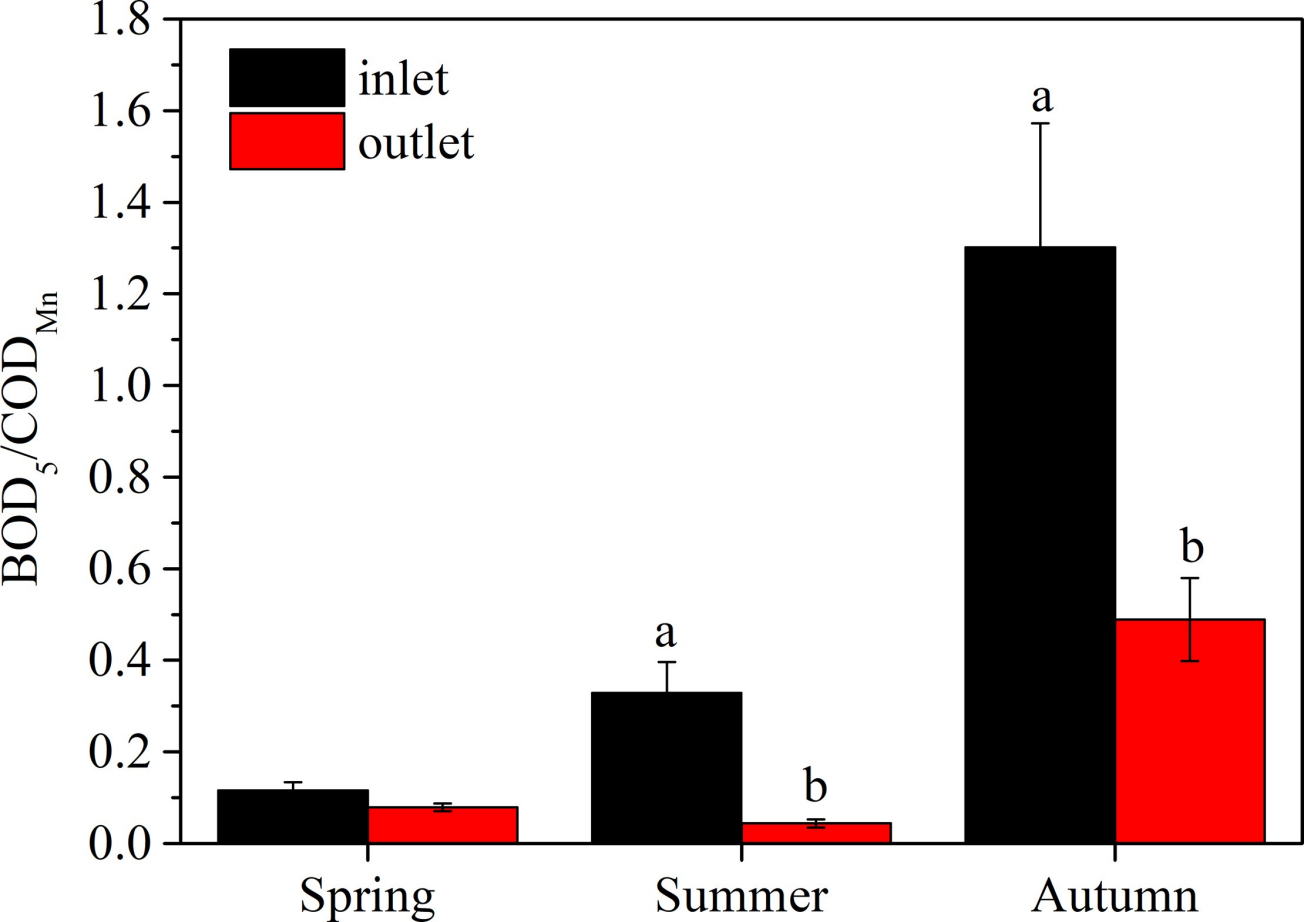
Seasonal variation in the ratio of BOD_5_/COD_Mn_ in the wetland inlet and outlet. There are non-significant differences between two error bars sharing the same letter.

The ratio of BOD_5_/COD_Mn_ in the inlet increased from spring to autumn and was greater than that in the outlet, except in spring. The highest ratios occurred in autumn, which were up to 1.30 and 0.33 for inlet and outlet, respectively (Fig 5).

### EAI assessment

The value of each indicator (water quality, plant landscape, acoustic and odour measurements) is listed in Table 4. The weights of the above indicators were 0.3, 0.3, 0.2 and 0.2, respectively, according to the Delphi method. The EAI of the inlet and outlet during different seasons is also shown in Table 4.

**Table 4:** Calculated indicators and EAI of the inlet and outlet during different seasons.

Site	Season	TSI	PLVHI	AEI	OI	EAI
**Inlet**	Spring	57.2	30	47	35	42.6
	Summer	58.0	58	83	68	65
	Autumn	57.5	90	74	52	69.5
**Outlet**	Spring	44.3	35	43	29	38.2
	Summer	58.1	72	67	73	67.0
	Autumn	48.2	93	72	43	65.4

## Discussion

### Current surface water purification efficiency

Fujin City is one of the key grain production bases of Northeast China. To maintain ecological health and sustainable agricultural development, both the central and local governments implement various projects for restoring farmland to wetlands, such as FNWP. Water quality is a primary issue in converting farmland to wetlands. Biodegradation of pollutants in surface water can be achieved using selected aquatic plants, which is definitively an appropriate method for long-term management in ecological and environmental restoration.

The wetland water quality monitoring results (Table 3, Fig 3) indicated that more efforts and improvements should be deployed. For example, BOD5 value is still a key parameter that can determine the level of organics, particularly the content of biodegradable organics in water [39]. BOD_5_/COD_Mn_ ratio could be used as an indicator of biodegradability improvement, where a higher value indicates better biodegradability [40]. Our results (Fig 5) indicated that biodegradability increased significantly when water was treated with wetlands. Therefore, considering that the existing removal efficiencies were not significantly high (Fig 4), higher efficiency could be expected when more plants are introduced into the wetland and longer water residence times are achieved. When adequate freshwater replenished in FNWP, the dilution effect would help microbes degrade organics better and prompt the performances of such plants [37]. In addition, TP is one of the most important pollutants for many freshwater wetlands, such a low removal efficiency in FNWP should be addressed, although the mean value of TP during our monitoring period was not too high to endanger the water quality.TP removal could be markedly improved with the planting of *M*. *spicatum* and *L*. *salicaria* (Table 1).

### Current aesthetic perception

The visual pollution of the wetland waterscape is an aesthetic issue and refers to the impacts of pollution that impair one's ability to enjoy a vista or view. Water pollution is a type of visual disturbance of human perception of the colour of the water [41].According to prior research to understand, after water purification which treated by natural and artificial approach. The colour of water has been change a subtle, a little bit clear and few of fish can be seen.

As a national wetland park and tourist destination, the current visual appearance of FNWP is not satisfactory for the present tourist demand. Aquatic plants (Table 1) are a purification tool for filtering pollutants in a water environment. Furthermore, aquatic plants are also a visual tool to create aquatic scenes with an ecological aesthetic [42]. Are the existing aquatic plants appropriately positioned within the aquatic landscape? Aquatic plants are a common sight to local residents and tourists. Our questionnaire suggested that the visual characteristics of the water’s appearance are crucial factors in assessing the ecological quality of the public's perception, for the PLVHI shared the same weight with the TSI (Tables 2 and 4). In many cases, the water quality of wetland restoration is based on the aesthetic aspect of a water environment. To some extent, aesthetic preferences strongly influence ecological work and the public’s acceptance of wetland management practices.

**Fig 6:**
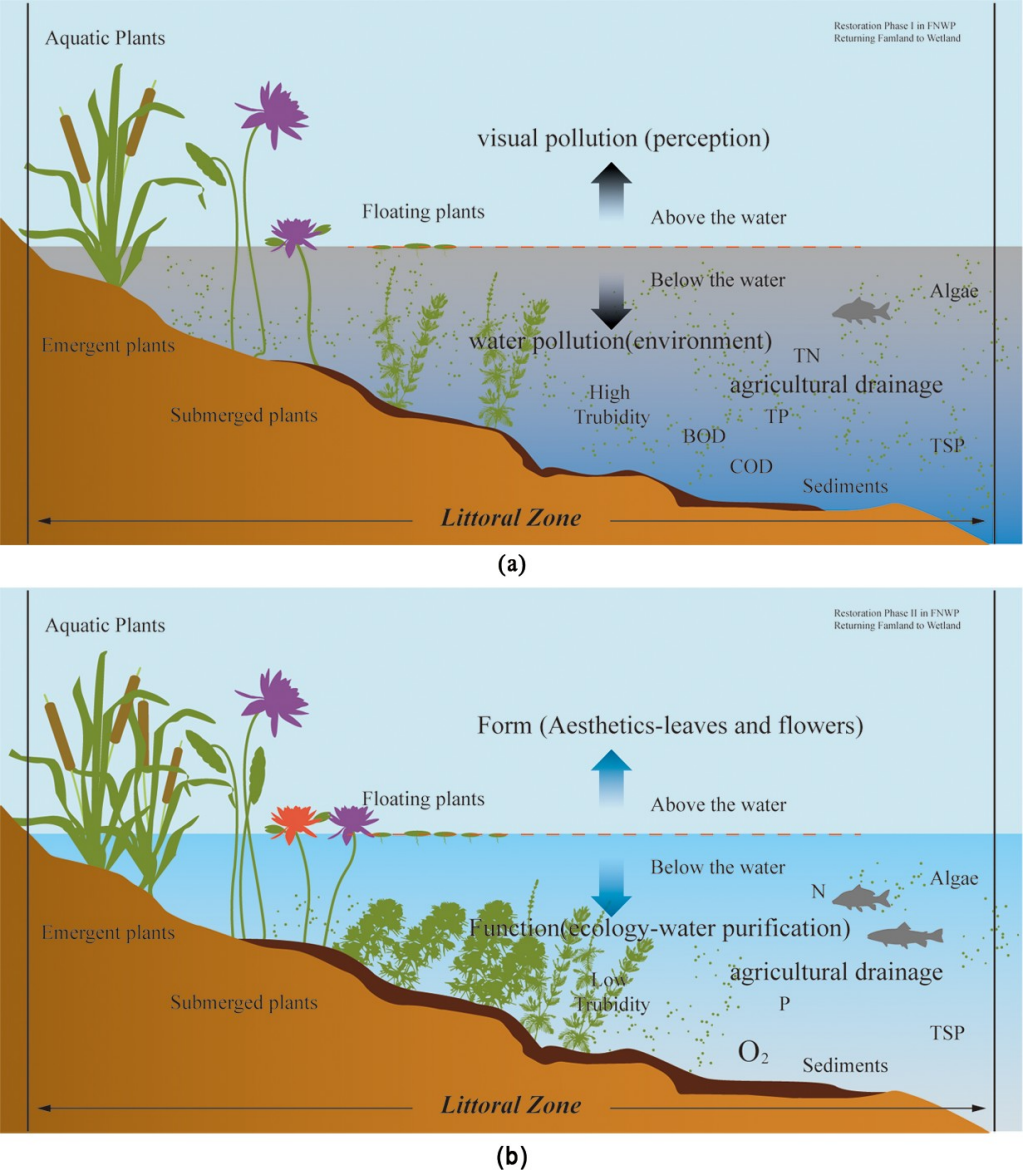
The comparative effects of using aquatic plants for agricultural drainage treatment. (a) Current status: low water transparency and many water pollutants, attributed to limited plant types, simple assemblages and insufficient plant amounts during the conversion of farmland to wetland; (b) desired status: high water transparency and fewer pollutants resulting from diverse and purification-effective plant arrangement, with more visual appeal.

In fact, aesthetic preference may have affected our behaviours and our understanding of the natural world. Ecologists and biologists rarely acknowledge the way beauty biases can affect research, although they may be significant and therefore worthy of discussing. From water pollution to visual pollution, this is the interdisciplinary research focus of ecological and aesthetic approaches to wetland restoration. The water purification using aquatic plants in agricultural wetland is gradually changing in these aspects. Water colour is brighter from dark to please the eye. Meanwhile, the odour of water smells better than before, not to smelly or stinky. Water environment is more pleasant for local residents and tourists to some degree. The holistic landscape ecology is acceptable than before during the stage of returning farmland to wetland in FNWP, especially as a national park for a long-term run (Fig 6).

### Interaction among aquatic plants, water and waterscape

Water can be evaluated as a feature of preferred landscapes. The waterscape can also be evaluated by two criteria. One is the shape of the water that surrounds plants; the other is the capacity of the water or the water level. These two visual factors of waterscapes affect people’s appreciation to some extent. What we studied above is an interdisciplinary area of the restoration of farmland to wetlands in China. This paper not only considered the purification of surface water but also the conservation of ecological aesthetics in the initial stages of wetland restoration. It also concurrently covers the different aspects of wetland wastewater treatment. Aquatic plants are functional plants that operate and interact with the aquatic environment. Therefore, the role of aquatic plants in surface water treatment is valuable for water purification (Table 3). Aquatic plants have an impact on waterscapes in landscape design by using an ecological and aesthetic approach. There is no denying that attaining a state of equilibrium among aquatic plants, aquatic-scenic design, and waterscape is more significant in wetland restoration. To better the synthetic assessment and management of such wetlands being restored from farmland, more water landscape and quality parameters should be monitored in future studies.

### Implication for aquatic plant management

The selection of species for restoring farmland to wetlands is critical. It is possible to reconcile the dual objectives of gradually increasing plant biomass to improve water purification and enhancing the aesthetic attraction by rationally arranging aquatic plants. For example, some species with high purification and aesthetic values (e.g. *L*. *salicaria*, *Canna indica*), have been successfully used in constructed wetlands [43,44].

According to the EAI results (Table 4), some aquatic plants are selected and planted with different functions. (1) Aquatic plants that suisee for the local characterises. (2) Aquatic plants that improve the water environment treatment for ecosystem (3) Aquatic plants that has aesthetic functions for landscape.

There could be there levels form water inlet to water outlet, submerge plants are used to purify the water as the first level. Floating plants and emergent plants are used to improve the visual aesthetic as the second level. Emergent plants are used to enhance the olfactory aesthetic as the third level. By the way, the arrangement of aquatic plants is not strict to follow the order, it will change follow the surroundings in groups.

Considering the important role of the plant landscape in the EAI, we emphasized the aquatic plants with water purification function and ecological aesthetic benefits. Based on the existing aquatic plants in FNWP, four management recommendations for arranged aquatic plants in the rebuilt wetland (Fig 6) are listed as follows:

1. Aesthetically improve the visual appeal of the waterscape. Aquatic plants maintain water clarity by preventing the re-suspension of bottom sediments. Water clarity changes subtly from low transparency to high transparency. Sustainable filtration is attained with aquatic plants that filter water.
2. Effectively improve the visual appeal by utilizing diverse plants. *N*. *nucifera*, *N*. *tetragona*, and *M*. *spicatum* have flowers or leaves that many people enjoy. Wetland plant arrangement is a proper way to enhance the visual hierarchy in waterscape design.
3. In addition to the original species, some native plants from the surrounding wetlands, such as *Scirpus validus*, could be introduced or transplanted to enhance the aesthetic and purification effects.
4. An endangered local carnivorous plant, *A*. *vesiculosa*, that has been listed under national first-class level protection needs further experimentation and monitoring to test what kinds of environmental factors affect its survival, and then better conserve it. Such a species is a good indicator of wetland condition because it disappeared in most parts of the Sanjiang Plain many years ago.

## Conclusions

We investigated and assessed the ecological aesthetic conditions of FNWP with seasonal variations. The surface water quality, which reflects ecological function and aesthetic value, could be improved after wetland treatment. However, the current plant landscape as well as the acoustic and odour conditions need further enhancement. Local diverse aquatic plants that can simultaneously remove pollutants and deliver a scenic solution are strongly recommended to be introduced to FNWP. This study highlighted the function of aquatic plants in surface water treatment to enhance ecological and environmental aesthetics, which will help to improve the aquatic environment by utilizing aquatic plants in waterscape design.
